# Age- and gender-specific reference intervals of TSH and free T4 in an iodine-replete area: Data from Korean National Health and Nutrition Examination Survey IV (2013–2015)

**DOI:** 10.1371/journal.pone.0190738

**Published:** 2018-02-01

**Authors:** So Young Park, Hae In Kim, Hyun-Kyung Oh, Tae Hyuk Kim, Hye Won Jang, Jae Hoon Chung, Myung-Hee Shin, Sun Wook Kim

**Affiliations:** 1 Division of Endocrinology and Metabolism, Department of Medicine, Thyroid Center, Samsung Medical Center, Sungkyunkwan University School of Medicine, Seoul, Korea; 2 Department of Social and Preventive Medicine, Sungkyunkwan University School of Medicine, Seoul, Korea; 3 Department of Medical Education, Sungkyunkwan University School of Medicine, Seoul, Korea; Boston University School of Medicine, UNITED STATES

## Abstract

**Background:**

Establishment of the reference interval of thyroid-stimulating hormone (TSH) is critical in the diagnosis of thyroid dysfunction and is affected by age, gender, iodine nutrition, and ethnicity. The aim of this study was to determine the reference intervals of TSH and free thyroxin (FT4) from a large, nationwide data of Korea where iodine intake is more than adequate.

**Methods:**

We analyzed data from the Korea National Health and Nutrition Examination Survey VI that measured serum TSH, FT4, and thyroid peroxidase antibody from 7,061 individuals (urinary iodine measurement in 6,565). Age- and gender-specific reference intervals were established from 95% confidence limits from the 2.5 to 97.5 percentile of TSH (log-transformed) and FT4 in reference populations.

**Results:**

The geometric mean of TSH was 2.16 ± 0.01 mIU/L, with the lowest value found in the middle aged group (2.04 ± 0.02 mIU/L) and higher values noted in age groups of 10–19 and over 70 years (2.38 ± 0.02 and 2.32 ± 0.07 mIU/L, respectively). The association of TSH and age was U-shaped. The overall reference interval of TSH was 0.59–7.03 mIU/L. Mean FT4 was 1.25 ± 0.003 ng/dL (16.09 ± 0.039 pmol/L), and it showed a small but continuous decrease after 20 years of age (P < 0.001). There was a significant positive correlation between TSH and urine iodine concentration (r = 0.154, P < 0.001).

**Conclusions:**

The reference interval of TSH in Korea, where iodine intake is above the requirement, was 0.59–7.03 mIU/L and showed U-shaped change with age, which was a similar pattern to iodine intake. The reference interval of FT4 was 0.92–1.60 ng/dL. The geometric mean and upper limit of TSH were higher than those of Western populations, reflecting the paramount importance of iodine intake on thyroid function.

## Introduction

Serum thyroid-stimulating hormone (TSH) is the most sensitive marker to evaluate individual thyroid functional status and is used as a screening test for identifying subjects with thyroid dysfunction [1]. Subclinical thyroid disease comprises subclinical hypothyroidism, defined as elevated TSH with normal free thyroxine (FT4), and subclinical hyperthyroidism, with decreased TSH and normal FT4 [2,3]. With the increased awareness of thyroid disorders and health check-ups, there are increased incidences of subclinical hypo- and hyperthyroidism [4,5]. In this clinical scenario, establishing a reference interval of TSH is critical for the diagnosis of subclinical thyroid functional disorders. However, the reference intervals of TSH are affected by many factors such as age, gender, ethnicity, iodine intake, body mass index (BMI), smoking, and the presence of thyroid autoantibody [6–8]. Current National Academy of Clinical Biochemistry (NACB) guidelines recommend that the reference intervals of TSH should be established from the 95% confidence limits of the log-transformed values of at least 120 thyroid peroxidase antibody (TPOAb)-negative, ambulatory, euthyroid subjects without goiter or family history of thyroid dysfunction [9].

Many studies have shown that the median and upper limit of TSH increase with age [10–12]. If we use the same reference interval of TSH in older patients as is younger patients, there is a chance of over-diagnosis of subclinical thyroid disease, which could lead to unnecessary treatment with levothyroxine in the older population.

Korea is known to be an iodine-replete area due to the popular nationwide dietary intake of iodine-rich seaweeds such as kelp and laver. Above-requirement or excessive iodine intake according to World Health Organization (WHO) epidemiological criteria was reported in preschool children as well as adults [13–16]. Although age and iodine are very important determinants in determination of the reference intervals of TSH and other thyroid hormone levels, there have been no such measurements performed according to age group and gender in Korea, where iodine intake is quite different from Western countries.

The Korea National Health and Nutrition Examination Surveys (KNHANES) is a nationwide cross-sectional survey to obtain national estimates of the health and nutritional status of Koreans from 1998 and is an ongoing surveillance system. KNHANES VI (2013–2015) introduced serum TSH, FT4, and TPOAb and thyroid disease-related items in the questionnaires. Thus, we sought to determine the reference intervals of TSH and FT4 from nationwide representative data of Korea.

In this article, we provide the age- and gender-specific reference intervals of TSH and FT4 from a large, nationwide, stratified dataset (KNHANES VI) from an iodine-replete area. In addition, we evaluated the association between iodine intake by age and TSH in this population.

## Methods

### Study subjects

The KNHANES surveys were designed to obtain national estimates of the health and nutritional status of Koreans from 1998 and are an ongoing surveillance system. KNHANES VI (2013–2015) was conducted using a stratified, multistage, clustered probability sampling design. In this data set, approximately 2,400 individuals (about one-third of the total sample) were selected each year between 2013 and 2015 using stratified subsampling and underwent measurements for serum TSH, FT4, and TPOAb. This subsample of KNHANES VI consisted of 7,061 individuals aged 10 years and older, weighted to represent the total Korean population. In this sample, urinary iodine (UI) in a spot urine sample was measured in 6,564 individuals. The participants responded to a questionnaire regarding family history of thyroid disease, personal history of thyroid disease, and drugs that could affect thyroid hormone level.

To define precise reference intervals of thyroid hormones, disease-free and reference populations were established. The disease-free population was selected from the total population after exclusion of individuals with known thyroid disease, family history of thyroid dysfunction, and current pregnancy. The reference population was selected from the disease-free population after exclusion of those with positive TPOAb, defined as ≥ 34.0 IU/ml, as provided by the kit manufacturer.

The study protocol was approved by the Institutional Review Board of the Korea Centers for Disease Control and Prevention. Written informed consent was obtained from all participants or parents/guardians.

### Data collection and laboratory methods

Data on demographic characteristics, personal medical history, and family history of thyroid diseases were collected by interview during the survey. Life style risk factors (i.e., smoking status) were based on self-reporting. Serum TSH, FT4, and TPOAb concentrations were measured by an electrochemiluminescence immunoassay. Serum TSH was measured using an E-TSH kit (Roche Diagnostics, Mannheim, Germany). Serum FT4 was measured by E-Free T4 kit (Roche Diagnostics, Mannheim, Germany). Serum TPOAb was measured by E-Anti-TPO kit (Roche Diagnostics, Mannheim, Germany). The results of TSH, FT4, and TPOAb met the specifications regarding accuracy, general chemistry, special immunology, and ligand of the quality control and quality assurance program of the College of American Pathologists. UI was measured using an inductively coupled plasma mass spectrometry device (ICP-MS; Perkin Elmer ICP-MS, Waltham, MA, USA). The laboratory that measured UI is enrolled in the “Ensuring the Quality of Urinary Iodine Procedures (EQUIP)” quality assurance program run by the Centers for Disease Control of the United States of America [17].

### Statistical analyses

We used SAS version 9.4 (SAS Institute, Cary, NC) to analyze complex sample survey data using the weights assigned to the individuals sampled in order to represent the nationwide population. The geometric mean ± standard error (SE) for TSH (log-transformed value), arithmetic mean ± SE for FT4, and median (interquartile range [IQR]) for UI were calculated as descriptive statistics using Proc Surveymeans. The 95% confidence limits from the 2.5 percentile to 97.5 percentile of TSH (log-transformed) and FT4 were obtained to establish reference intervals. Regression analysis using Proc Surveyreg was used to compare the differences in TSH and FT4 between the age groups and genders. Correlation coefficients using Proc Corr were adjusted for age, gender, BMI, and smoking status and were calculated to estimate the relationship between TSH and UI concentration. A p-value < 0.05 was considered significant.

## Results

### Baseline characteristics of the study subjects

Among 7,061 individuals who underwent thyroid function evaluation as a part of KNHANES VI, we excluded 705 individuals who had a prior history of thyroid disease (n = 179, 2.2%) or thyroid cancer (n = 44, 0.5%), family history (n = 346, 4.8%), or were pregnant (n = 22, 0.3%) to establish a disease-free population. Among the disease-free population of 6,356 individuals, we excluded individuals who had a positive TPO Ab result (n = 370, 5.7%). Finally, the reference population consisted of 5,987 individuals. The mean age of the reference population was 37.0 ± 0.2 years, and the proportion of males was 51.8%. The detailed characteristics of each population group are shown in Table 1.

**Table 1:** Characteristics of total, disease-free, and reference populations.

	Total population			Disease-free population^a^			Reference population^b^		
	Total	Male	Female	Total	Male	Female	Total	Male	Female
Number of individuals, n	7,061	3,493	3,568	6,356	3,216	3,140	5,987	3,104	2,883
Age, years	37.6 ± 0.2	37.7 ± 0.2	37.5 ± 0.2	37.4 ± 0.2	37.6 ± 0.2	37.2 ± 0.3	37.0 ± 0.2	37.3 ± 0.2	36.6 ± 0.3
Known thyroid disease, n	179 (2.2)	26 (0.8)	153 (4.1)						
Known thyroid cancer, n	44 (0.5)	7 (0.1)	37 (1.0)						
Family history, n	346 (4.8)	134 (4.0)	212 (5.9)						
TSH, mIU/L	2.14 ± 0.01	2.1 ± 0.01	2.19 ± 0.02	2.20 ± 0.01	2.11 ± 0.01	2.29 ± 0.02	2.16 ± 0.01	2.09 ± 0.01	2.24 ± 0.02
FT4, ng/dL	1.24 ± 0.003	1.29 ± 0.004	1.20 ± 0.005	1.24 ± 0.003	1.28 ± 0.004	1.20 ± 0.005	1.25 ± 0.003	1.29 ± 0.004	1.2 ± 0.005
TPOAb, IU/mL	34.6 ± 3.2	21.3 ± 2.8	48.4 ± 5.7	27.3 ± 2.5	19.5 ± 3.0	35.8 ± 4.0	7.7 ± 0.1	7.6 ± 0.1	7.9 ± 0.1
TPOAb positivity^c^, n	503 (7.1)	147 (4.3)	356 (10.1)	369 (5.7)	112(3.4)	257 (8.2)			
UI, ug/L, median	290.5 (157.2–667.6)	287.0 (160.9–649.9)	298.6 (151.6–694.6)	292.1 (157.3–670.1)	287.5 (160.9–658.5)	300 (151.6–691.4)	292.3 (158.7–670.8)	287.9 (161.9–654.1)	299.8 (152.8–701.6)
Smoking status, n	1,349 (27.1)	1,158 (43.1)	191 (6.8)	1,270 (27.8)	1,096 (42.9)	174 (7.1)	1,214 (28.3)	1,056 (42.9)	158 (7.0)
BMI, kg/m^2^	23.5 ± 0.1	24.1 ± 0.1	22.8 ± 0.1	23.5 ± 0.1	24.1 ± 0.1	22.7 ± 0.1	23.5 ± 0.1	24.0 ± 0.1	22.6 ± 0.1

Mean ± Standard error, number (%), except geometric mean ± standard error for TSH and median (interquartile range) for UI.

^a^ Disease-free population was defined as individuals without known thyroid disease, family history of thyroid dysfunction, or current pregnancy.

^b^ Reference population was defined as individuals without known thyroid disease, family history of thyroid dysfunction and positive TPOAb, or current pregnancy.

^c^ TPOAb positivity was defined as ≥ 34.0 IU/ml, as provided by the kit manufacturer.

TSH, thyroid-stimulating hormone; FT4, free thyroxine; TPO Ab, thyroid peroxidase antibody; UI, urinary iodine; BMI, body mass index.

### TSH by age and gender

The geometric mean ± SE of TSH in the total population was 2.14 ± 0.01 mIU/L (Table 2), with the lowest value found in the age group of 40–49 years (2.04 ± 0.03 mIU/L) and higher values noted in age groups of 10–19 and over 70 years (2.33 ± 0.02 and 2.37 ± 0.07 mIU/L, respectively). In the reference population, the geometric mean TSH was 2.16 ± 0.01 mIU/L. The geometric mean of TSH in the reference population was lower in the middle aged group (2.04 ± 0.02 mIU/L) and higher in age groups of 10–19 and over 70 years (2.38 ± 0.02 and 2.32 ± 0.07 mIU/L, respectively). Thus, a U-shaped association was observed between age and TSH concentration.

**Table 2:** Thyroid-stimulating hormone ^a^ concentration by age and gender.

	Total population					Disease-free population^b^					Reference population^c^				
	n	Mean^d^	Percentile			n	Mean^d^	Percentile			n	Mean^d^	Percentile		
			2.5	Median	97.5			2.5	Median	97.5			2.5	Median	97.5
**All**															
10-19yr	1,135	2.33	0.58	2.42	7.37	1,084	2.36	0.61	2.45	7.44	1,057	2.38	0.63	2.45	7.27
20-29yr	1,116	2.15	0.62	2.25	6.24	1,007	2.18	0.66	2.26	6.46	970	2.17	0.67	2.25	6.05
30-39yr	1,197	2.05	0.54	2.12	6.87	1,032	2.10	0.62	2.12	6.67	988	2.05	0.61	2.07	6.42
40-49yr	1,197	2.04	0.50	2.14	8.38	1,038	2.13	0.59	2.15	8.30	975	2.04	0.58	2.09	6.20
50-59yr	1,221	2.22	0.38	2.38	9.78	1,098	2.23	0.45	2.37	8.80	993	2.20	0.49	2.30	8.11
60-69yr	1,083	2.09	0.34	2.24	7.88	991	2.18	0.54	2.24	7.87	904	2.17	0.56	2.21	7.77
70yr<	112	2.37	0.34	2.29	7.50	106	2.36	0.43	2.29	6.88	100	2.32	0.42	2.28	6.68
Total	7,061	2.14	0.51	2.25	7.57	6,356	2.20	0.57	2.26	7.51	5,987	2.16	0.59	2.23	7.03
**Male**															
10-19yr	591	2.40	0.72	2.46	6.84	566	2.44	0.75	2.47	6.91	558	2.44	0.75	2.47	6.92
20-29yr	534	2.15	0.66	2.24	6.23	497	2.15	0.67	2.24	6.35	487	2.13	0.66	2.22	6.09
30-39yr	581	2.00	0.65	2.06	5.88	512	2.02	0.69	2.06	6.04	498	2.00	0.68	2.04	6.05
40-49yr	591	1.95	0.52	2.02	6.78	523	1.99	0.59	1.99	6.42	508	1.94	0.59	1.97	5.96
50-59yr	596	2.12	0.55	2.13	8.11	550	2.08	0.51	2.09	7.35	522	2.06	0.53	2.08	7.02
60-69yr	541	2.05	0.51	2.13	8.95	512	2.06	0.52	2.13	7.95	479	2.07	0.53	2.13	7.35
70yr<	59	2.13	0.11	2.04	7.85	56	2.23	0.29	2.07	6.64	52	2.18	0.28	2.04	6.53
Total	3,493	2.10	0.58	2.17	6.96	3,216	2.11	0.61	2.16	6.9	3,104	2.09	0.62	2.14	6.57
**Female**															
10-19yr	544	2.26	0.56	2.36	7.49	518	2.27	0.56	2.38	7.50	499	2.31	0.57	2.39	7.47
20-29yr	582	2.15	0.53	2.25	6.20	510	2.21	0.62	2.27	6.39	483	2.22	0.67	2.26	6.00
30-39yr	616	2.10	0.47	2.22	7.23	520	2.19	0.50	2.24	7.11	490	2.12	0.50	2.17	6.54
40-49yr	606	2.15	0.40	2.23	11.80	515	2.32	0.55	2.28	11.86	467	2.16	0.55	2.20	7.20
50-59yr	625	2.33	0.06	2.63	11.99	548	2.42	0.20	2.64	9.79	471	2.38	0.42	2.56	9.19
60-69yr	542	2.12	0.18	2.36	7.76	479	2.30	0.56	2.37	7.87	425	2.26	0.56	2.28	7.87
70yr<	53	2.64	0.51	2.62	6.88	50	2.52	0.46	2.42	6.89	48	2.48	0.44	2.42	6.80
Total	3,568	2.19	0.37	2.34	8.18	3,140	2.29	0.54	2.36	8.07	2,883	2.24	0.56	2.31	7.43

^a^ Thyroid-stimulating hormone (mill international units per liter).

^b^ Disease-free population was defined as individuals without known thyroid disease, family history of thyroid dysfunction, or current pregnancy.

^c^ Reference population was defined as individuals without known thyroid disease, family history of thyroid dysfunction and positive thyroid peroxidase antibody, and current pregnancy.

^d^ The mean was geometric mean, calculated from logarithmically transformed values.

**Fig 1:**
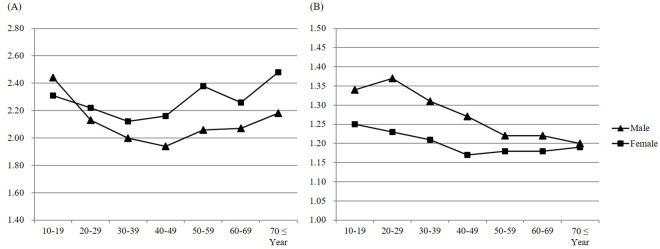
Thyroid-stimulating hormone and free thyroxine by age and gender in the reference population. (A) Geometric mean of TSH (mIU/L) by age. (B) Mean of FT4 (ng/dL) by age.

The geometric mean of TSH was significantly higher in females (2.24 ± 0.02 mIU/L) than males (2.09 ± 0.01 mIU/L) in the reference population (P < 0.001), but showed marginal difference in the total population (female vs. male: 2.19 ± 0.02 vs 2.10 ± 0.01 mIU/L, P = 0.056). Age- and gender-specific geometric means of TSH are shown in Fig 1.

### Age- and gender-specific reference intervals of TSH

The overall reference interval of TSH in the reference population was 0.59–7.03 mIU/L and was wider in females (0.56–7.43 mIU/L) than males (0.62–6.57 mIU/L) (Table 2). The reference interval of TSH had no significant association with age (P for trend = 0.09). The upper reference limit of TSH was the lowest in those aged 20–29 years (6.04 mIU/L) and highest in those aged 50–59 years (8.11 mIU/L). In males, it was lowest in the age group of 40–49 years (5.96 mIU/L) and highest in the group 60–69 years (7.35 mIU/L); in females, the lowest upper reference TSH level was found in 20-29-year-olds (5.97 mIU/L) and the highest was seen in 50-59-year-olds (9.19 mIU/L). The lower reference limit of TSH was the lowest in the age group over 70 years (0.42 mIU/L) and highest in the group 20–29 years (0.67 mIU/L).

### Age- and gender-specific reference intervals of FT4

In the reference population, mean FT4 was 1.25 ± 0.003 ng/dL (16.09 ± 0.039 pmol/L) (Table 3). After age 20 years, FT4 decreased significantly with age (P for trend < 0.0001). Mean FT4 in males (1.29 ± 0.003 ng/dL, 16.60 ± 0.039 pmol/L) was significantly higher than that in females (1.20 ± 0.005 ng/dL, 15.44 ± 0.064 pmol/L) (P < 0.0001). The overall reference range of FT4 was 0.92–1.60 ng/dL (11.84 ± 20.59 pmol/L). The tendency of mean FT4 in the reference population was similar to that of the total population.

**Table 3:** Free thyroxine^a^ concentration by age and gender.

	Total population					Disease-free population^b^					Reference population^c^				
	n	Mean	Percentile			n	Mean	Percentile			n	Mean	Percentile		
			2.5	Median	97.5			2.5	Median	97.5			2.5	Median	97.5
**All**															
10-19yr	1,135	1.30	0.97	1.28	1.69	1,084	1.30	0.98	1.28	1.69	1,057	1.29	0.98	1.28	1.68
20-29yr	1,116	1.31	0.99	1.29	1.69	1,007	1.31	1.00	1.29	1.66	970	1.31	1.00	1.30	1.67
30-39yr	1,197	1.26	0.95	1.24	1.61	1,032	1.26	0.95	1.24	1.60	988	1.26	0.95	1.24	1.60
40-49yr	1,197	1.22	0.89	1.20	1.58	1,038	1.22	0.90	1.20	1.56	975	1.23	0.92	1.21	1.56
50-59yr	1,221	1.20	0.86	1.18	1.58	1,098	1.20	0.89	1.18	1.58	993	1.20	0.89	1.19	1.55
60-69yr	1,083	1.20	0.87	1.18	1.57	991	1.19	0.87	1.19	1.55	904	1.20	0.87	1.19	1.55
70yr<	112	1.20	0.82	1.18	1.51	106	1.20	0.79	1.18	1.50	100	1.19	0.78	1.18	1.50
Total	7,061	1.24	0.91	1.22	1.62	6,356	1.24	0.91	1.23	1.61	5,987	1.25	0.92	1.23	1.60
**Male**															
10-19yr	591	1.35	1.02	1.32	1.74	566	1.34	1.02	1.31	1.72	558	1.34	1.02	1.31	1.73
20-29yr	534	1.37	1.06	1.36	1.74	497	1.37	1.05	1.36	1.71	487	1.37	1.05	1.36	1.72
30-39yr	581	1.30	0.98	1.29	1.61	512	1.31	0.98	1.29	1.61	498	1.31	0.98	1.29	1.61
40-49yr	591	1.27	0.93	1.26	1.59	523	1.27	0.95	1.26	1.58	508	1.27	0.96	1.26	1.59
50-59yr	596	1.22	0.90	1.20	1.59	550	1.22	0.90	1.20	1.59	522	1.22	0.91	1.20	1.56
60-69yr	541	1.21	0.89	1.20	1.56	512	1.21	0.89	1.21	1.55	479	1.22	0.90	1.21	1.54
70yr<	59	1.21	0.60	1.21	1.50	56	1.20	0.57	1.20	1.49	52	1.20	0.56	1.20	1.49
Total	3,493	1.29	0.94	1.27	1.64	3,216	1.28	0.95	1.27	1.63	3,104	1.29	0.96	1.27	1.63
**Female**															
10-19yr	544	1.25	0.94	1.23	1.63	518	1.25	0.94	1.23	1.64	499	1.25	0.95	1.23	1.62
20-29yr	582	1.23	0.95	1.23	1.54	510	1.23	0.97	1.23	1.54	483	1.23	0.97	1.23	1.54
30-39yr	616	1.21	0.92	1.19	1.55	520	1.21	0.93	1.19	1.50	490	1.21	0.94	1.19	1.50
40-49yr	606	1.17	0.83	1.15	1.52	515	1.16	0.85	1.15	1.51	467	1.17	0.89	1.15	1.51
50-59yr	625	1.19	0.84	1.15	1.55	548	1.18	0.87	1.14	1.52	471	1.18	0.86	1.16	1.51
60-69yr	542	1.18	0.85	1.17	1.61	479	1.18	0.84	1.17	1.55	425	1.18	0.84	1.18	1.56
70yr<	53	1.18	0.91	1.16	1.52	50	1.19	0.91	1.16	1.53	48	1.19	0.91	1.16	1.45
Total	3,568	1.20	0.87	1.18	1.58	3,140	1.20	0.89	1.18	1.55	2,883	1.20	0.90	1.19	1.54

^a^ Free thyroxine concentrations (nanograms per deciliter).

^b^ Disease-free population was defined as individuals without known thyroid disease, family history of thyroid dysfunction, or current pregnancy.

^c^ Reference population was defined as individuals without known thyroid disease, family history of thyroid dysfunction and positive thyroid peroxidase antibody, or current pregnancy.

### Urine iodine and TSH

**Fig 2:**
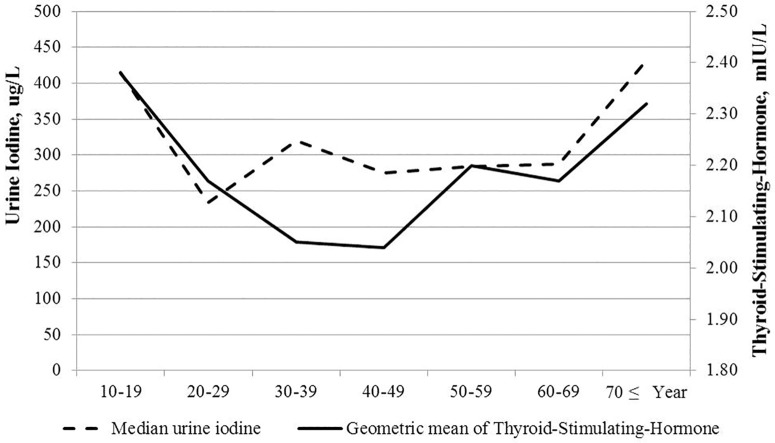
Relation of urine iodine concentration to thyroid-stimulating hormone.

In the reference population, the median UI (IQR) was 292.3 (158.7–670.8) ug/L, which is classified as above the required iodine intake according to WHO criteria. It was lower in the age groups of 20–29 years and 40–49 years (233.6 [138.3–514.8] and 275.0 [154.9–621.8] ug/L) and higher in age groups of 10–19 and over 70 years (414.7 [204.4–947.7] and 431.4 [140.0–984.7] ug/L, respectively) (Table 4). After adjusting for age, gender, BMI, and smoking status, there was a significant positive correlation between TSH and UI concentration (r = 0.154, P < 0.001). We also analyzed the association between TSH and UI by dividing the reference population into 3 groups according to age: younger than 30 years, between 30 and 50 years, and older than 50 years. The correlation coefficient (r) between TSH and UI in the groups was 0.185 (p = 0.008), 0.150 (P = 0.031), and 0.182 (P < 0.001), respectively. Accordingly, the curve of UI (median) by age showed very similar U-shaped appearance to that of TSH by age, suggesting iodine’s effect on TSH in this population (Fig 2).

**Table 4:** Urine iodine^a^ concentration by age and gender in the reference population.

	Total population			Disease-free population^b^			Reference population^c^		
	n	Median	IQR	n	Median	IQR	n	Median	IQR
**All**									
10-19yr	1,009	403.6	(204.9, 932.2)	968	415.7	(210.1, 946.9)	943	414.7	(204.4, 947.7)
20-29yr	1,006	237.7	(142.6, 517.7)	911	233.7	(139.1, 516.7)	875	233.6	(138.3, 514.8)
30-39yr	1,084	311.3	(160.2, 668.2)	943	317.9	(160.7, 684.2)	904	319.6	(161.4, 683.1)
40-49yr	1,101	273.9	(151.0, 622.1)	964	275.7	(155.1, 618.3)	903	275.5	(154.9, 621.8)
50-59yr	1,196	284.5	(150.4, 646.8)	1,078	284.5	(149.8, 643.4)	974	284.6	(157.0, 650.4)
60-69yr	1,058	288.7	(141.9, 817.4)	970	287.9	(138.7, 792.7)	886	287.9	(146.7, 776.7)
70yr<	110	428.4	(137.7, 977.4)	104	424.1	(137.5, 959.2)	98	431.4	(140.0, 984.7)
Total	6,564	290.5	(157.2, 667.6)	5,938	292.1	(157.3, 670.1)	5,583	292.3	(158.7, 670.8)
**Male**									
10-19yr	569	399.5	(204.5, 922.5)	545	404.8	(210.0, 949.1)	537	403.5	(204.5, 946.6)
20-29yr	514	234.3	(146.5, 504.8)	478	234.7	(146.3, 514.7)	468	238.0	(144.8, 507.0)
30-39yr	556	322.7	(164.6, 649.2)	496	322.8	(164.2, 662.3)	482	325.2	(168.2, 662.5)
40-49yr	569	270.3	(158.8, 618.2)	512	271.0	(160.0, 620.3)	497	271.2	(158.9, 612.3)
50-59yr	584	267.7	(157.3, 642.7)	540	266.6	(159.1, 643.9)	512	265.2	(160.3, 637.5)
60-69yr	527	276.0	(139.1, 755.0)	500	272.0	(138.6, 753.5)	468	273.8	(149.6, 736.8)
70yr<	59	342.4	(92.4, 886.7)	56	349.3	(93.2, 921.2)	52	358.0	(89.0, 953.5)
Total	3,378	287.0	(160.9, 649.9)	3,127	287.5	(160.9, 658.5)	3,016	287.9	(161.9, 654.1)
**Female**									
10-19yr	440	413.0	(205.1, 936.6)	423	432.3	(211.8, 940.5)	406	430.1	(200.6, 948.9)
20-29yr	492	239.1	(136.0, 561.1)	433	229.6	(132.3, 536.5)	407	229.3	(132.0, 520.6)
30-39yr	528	295.9	(152.0, 757.0)	447	298.9	(156.7, 805.8)	422	298.8	(158.1, 799.1)
40-49yr	532	277.9	(140.1, 640.7)	452	281.4	(142.9, 613.3)	406	280.9	(143.0, 656.2)
50-59yr	612	314.4	(141.0, 652.5)	538	311.9	(140.0, 641.2)	462	314.4	(140.5, 663.5)
60-69yr	531	311.9	(142.2, 863.4)	470	308.7	(138.6, 834.6)	418	309.8	(144.4, 818.2)
70yr<	51	450.9	(148.8, 1190.0)	48	436.7	(148.1, 1114.5)	46	438.2	(150.8, 1107.5)
Total	3,186	298.6	(151.6, 694.6)	2,811	299.9	(151.2, 688.8)	2,567	299.8	(152.8, 701.6)

^a^ Urine iodine concentration (median, micrograms per liter).

^b^ Disease-free population was defined as individuals without known thyroid disease, family history of thyroid dysfunction, or current pregnancy.

^c^ Reference population was defined as individuals without known thyroid disease, family history of thyroid dysfunction and positive thyroid peroxidase antibody, or current pregnancy.

IQR, interquartile range.

## Discussion

This study presented the age- and gender-specific reference intervals of TSH and FT4 from the large, nationwide survey dataset of KNHANES VI (2013–2015). The results demonstrated that TSH has a U-shaped association with age and is higher in females. In addition, there was a strong association between UI by age and TSH by age. In contrast, FT4 decreased continuously after 20 years of age and was higher in males than in females. To our knowledge, this is the first study to establish reference intervals of TSH and FT4 from a large, nationwide study in Korea, where iodine intake is reported to be above-requirement or excessive according to WHO epidemiological criteria.

Previous studies from Korea based on two regional population-based cohorts reported similar results to our report [18,19]. In one study from an Ansung cohort (n = 3,399, adults between 40 and 70 years old), mean FT4 was 0.99 ± 0.14 ng/dL, and mean TSH was 2.53 ± 3.31 mIU/L. In the other study from the Korean Longitudinal Study on Health and Aging (KLoSHA, n = 940), which consisted of elderly subjects over 65 years old, mean FT4 was 1.22 ± 0.38 ng/dL, and mean TSH was 3.49 ± 7.12 mIU/L. Only in males of the Ansung cohort, TSH increased and FT4 decreased with age; age was not associated with TSH and FT4 in the other subjects. The other study from Korea, based on routine health examination data, also reported a higher reference interval of TSH from 0.73 to 7.06 mIU/L [20]. However, the data were all regional or institutional rather than nationwide standardized data like our study.

There are some distinctive differences between our report and other published reports. First, TSH in this study was apparently higher than that of other reports from Western populations. The geometric mean and overall upper reference limit of TSH were 2.16 and 7.03 mIU/L, respectively, in our study, whereas they were 1.40 and 4.12 mIU/L in the National Health and Nutrition Examination Survey (NHANES III) from the United States of America [10]. One possible explanation for these differences is related to the different iodine intake status of Korea, which was classified to be above-requirement or excessive in previous reports and also in this study. In other regions with sufficient iodine supply such as North America and East Asia [21,22], concentrations and reference limits of TSH tend to be higher than those in regions with iodine deficiency, such as Europe [23,24]. Similarly, in NHANES III, higher UI/Cr excretion was significantly related to higher TSH concentration [10]. These findings are consistent with some reports showing that distribution of TSH shifted toward the right after improved iodine supply [25,26]. However, this reason is not sufficient to explain these apparent differences, because our results were much higher than those in similar iodine intake regions. Even in an iodine-excessive area in China where the median UI of schoolchildren was 650.9 ug/L, the geometric mean and reference range of TSH were 1.90 and 0.59–5.98 mIU/L, respectively [27]. Thus, as recently suggested, hereditary and genetic influences on the set-point of thyroid hormone might be additional reasons for the high upper limits of this study. A few studies have suggested that the negative feedback set points for FT4 and TSH might be genetically determined [28]. Other reports suggested that polymorphisms in thyroid hormone pathway genes might be related with increased TSH concentration [29,30]. Further studies will be required to validate a genetic basis of thyroid hormone regulation.

Second, we did not show an increase of TSH with age but a U-shaped association. This U-shaped trend of TSH with age, with values lower in the middle age group and higher younger and older ages, was very similarly seen in UI concentration in this study. There was a significant correlation between UI by age and TSH by age. Therefore, we speculate that differences of iodine intake with age group affected TSH, as previously stated, and could offset the increase in TSH according to age reported in other reports [10,12,31]. However, as UI by age and TSH by age do not show a correlation in those aged 30–39 years, the change of TSH according to UI should be interpreted with caution. Further studies will be needed to more accurately clarify the association between TSH and iodine status.

Finally, continuous decrease of FT4 with age was observed after 20 years of age. This result is in accord with other studies about FT4, showing either no change or a minimal decrease in FT4 with age [10], although some reports suggested that the differences were too small to have clinical significance [31]. Recently, there were some reports showing that increased TSH and decreased free thyroid hormone are associated with prolonged life span in some populations [12,32]. These reports support the suggestion that age-specific reference ranges of TSH should be set in order to avoid over-treatment.

Most clinicians would agree to the need to establish reference intervals of thyroid hormones with consideration of age, gender, ethnicity, and iodine intake. However, they still use the values provided by the guidelines or commercial kits manufacturers because of the lack of regional or population data. Therefore, we hope that the reference intervals of TSH and FT4 provided in this study will be used as reference intervals in clinical practice in Korea and other areas where iodine intake is similar. Our findings show that the widely used upper limit of TSH of 4.5 or 5.0 mIU/L might be too low to be applicable where iodine intake is above-requirement and thus could lead to overestimates of subclinical hypothyroidism.

The limitation of this study is that the presence of goiter was not obtained during the survey by ultrasound or physical examinations but only by questionnaire. However, the similarity of the reference interval of this study to a previous report from Korea where the presence of goiter was checked by ultrasound support the solidarity of our study data [20]. In the previous study (n = 5,778), TSH RI obtained using ultrasound findings was 0.73–7.06 mIU/L, which is almost identical to our result. Thyroglobulin Ab (TgAb) also could not be obtained in this survey. However, it is known that the presence of TgAb alone does not correlate with abnormal TSH level [33]. Especially in iodide sufficient areas, it is not usually necessary or cost-effective to order both TPOAb and TgAb, because TPOAb-negative patients with detectable TgAb rarely display thyroid dysfunction [9]. Therefore, we cautiously speculate that the presence or absence of goiter or TgAb would not lead to a significant impact on our overall results. In addition, the KNHANES is cross-sectional and therefore does not show individual changes over time according to iodine intake and so cannot prove the relationships between iodine intake and TSH and/or FT4. Finally, TSH and FT4 were measured by one type of kit, even though the results met the specifications of the quality assurance program of the College of American Pathologists. Further studies are needed to evaluate the compatibility of this study’s results to results measured by different kits.

In summary, we present the reference intervals of TSH and FT4 from KNHANES VI data in Korea, where iodine intake is above-requirement. The reference interval of TSH was 0.59–7.03 mIU/L and showed U-shaped change with age, similar to the pattern of iodine intake. The reference interval of FT4 was 0.92–1.60 ng/dL and showed continuous decrease with age after 20 years. The geometric mean and upper limit of TSH were higher than those of Western populations, reflecting the paramount importance of iodine intake on thyroid function [34].
